# Tetramer Enrichment Reveals the Presence of Phenotypically Diverse Hepatitis C Virus-Specific CD8^+^ T Cells in Chronic Infection

**DOI:** 10.1128/JVI.02242-14

**Published:** 2014-12-16

**Authors:** Katja Nitschke, Tobias Flecken, Julia Schmidt, Emma Gostick, Matthias Marget, Christoph Neumann-Haefelin, Hubert E. Blum, David A. Price, Robert Thimme

**Affiliations:** a Department of Medicine II, University Hospital Freiburg, Freiburg, Germany; b Faculty of Biology, University of Freiburg, Freiburg, Germany; c Spemann Graduate School of Biology and Medicine, University of Freiburg, Freiburg, Germany; d Institute of Infection and Immunity, Cardiff University School of Medicine, Cardiff, United Kingdom; e Institute of Transfusion Medicine, University Hospital Hamburg-Eppendorf, Hamburg, Germany

## Abstract

Virus-specific CD8^+^ T cells are rarely detectable *ex vivo* by conventional methods during chronic hepatitis C virus (HCV) infection. In this study, however, we were able to detect and characterize HCV-specific CD8^+^ T cells in all chronically HCV genotype 1a-infected, HLA-A*02:01-positive patients analyzed by performing major histocompatibility complex (MHC) class I tetramer enrichment. Two-thirds of these enriched HCV-specific CD8^+^ T-cell populations displayed an effector memory phenotype, whereas, surprisingly, one-third displayed a naive-like phenotype despite ongoing viral replication. CD8^+^ T cells with an effector memory phenotype could not expand *in vitro*, suggesting exhaustion of these cells. Interestingly, some of the naive-like CD8^+^ T cells proliferated vigorously upon *in vitro* priming, whereas others did not. These differences were linked to the corresponding viral sequences in the respective patients. Indeed, naive-like CD8^+^ T cells from patients with the consensus sequence in the corresponding T-cell epitope did not expand *in vitro*. In contrast, in patients displaying sequence variations, we were able to induce HCV-specific CD8^+^ T-cell proliferation, which may indicate infection with a variant virus. Collectively, these data reveal the presence of phenotypically and functionally diverse HCV-specific CD8^+^ T cells at very low frequencies that are detectable in all chronically infected patients despite viral persistence.

**IMPORTANCE** In this study, we analyzed CD8^+^ T-cell responses specific for HLA-A*02:01-restricted epitopes in chronically HCV-infected patients, using MHC class I tetramer enrichment. Importantly, we could detect HCV-specific CD8^+^ T-cell populations in all patients. To further characterize these HCV-specific CD8^+^ T-cell populations that are not detectable using conventional techniques, we performed phenotypic, functional, and viral sequence analyses. These data revealed different mechanisms for CD8^+^ T-cell failure in HCV infection, including T-cell exhaustion, viral escape, and functional impairment of naive-like HCV-specific CD8^+^ T cells.

## INTRODUCTION

Low numbers of naive virus-specific CD8^+^ T cells circulate in the blood of healthy individuals (1, 2). After encountering cognate antigen, for example, during acute hepatitis C virus (HCV) infection, these virus-specific CD8^+^ T cells are primed to expand clonally and gain effector functions. In the majority of HCV-infected patients, however, viral evasion strategies impede the immune response and persistent infection ensues. The exact mechanisms that contribute to HCV-specific CD8^+^ T-cell failure are not completely understood, but growing evidence suggests that T-cell exhaustion and viral escape are primary contributors (3). Distinct phenotypic characteristics within the HCV-specific CD8^+^ T-cell compartment accompany both of these processes. In particular, exhausted CD127^−^ CD8^+^ T cells with an effector memory phenotype coexpress high levels of inhibitory receptors, such as programmed cell death-1 (PD-1) and target consensus epitopes, whereas CD127^+^ CD8^+^ T cells tend to express low levels of inhibitory receptors and target variant epitopes (4–7). It is important to note, however, that HCV-specific CD8^+^ T-cell populations cannot be detected directly *ex vivo* in the majority of chronically infected patients (2, 8). Consequently, such phenotypic studies have been limited by technological constraints to small numbers of patients. Recently, an *ex vivo* enrichment protocol based on the use of major histocompatibility complex (MHC) class I tetramers that allows the detection and characterization of rare antigen-specific CD8^+^ T-cell populations as well as an estimation of their frequency has been reported (1, 2). Using this approach, we previously quantified functionally competent naive HCV-specific CD8^+^ T cells in healthy donors (2). Here, we used a similar experimental design to analyze HCV-specific CD8^+^ T cells that could not be detected by conventional tetramer staining during chronic HCV genotype 1a infection. In this study, we found HCV-specific CD8^+^ T cells in all patients tested as well as a high proportion of naive-like HCV-specific CD8^+^ T cells in some patients. However, the proliferative capability of these cells was intact only in patients who displayed sequence variations in the corresponding viral epitopes. In contrast, the presence of consensus viral sequences was associated with an impaired proliferative capability, suggesting that in these patients a functional impairment of naive-like HCV-specific CD8^+^ T cells may contribute to HCV-specific CD8^+^ T-cell failure.

## MATERIALS AND METHODS

### Subjects.

Seventeen HLA-A*02:01-positive (HLA-A*02:01^+^) subjects with chronic HCV genotype 1a infection (Table 1) attending the University Hospital of Freiburg were included in the study. In addition, 12 HLA-A*02:01^+^ healthy individuals were included. Written informed consent was obtained in all cases, and the study was conducted in accordance with federal guidelines, local ethics committee regulations, and the Declaration of Helsinki (1975). Approval was obtained from the ethics committee of the Albert-Ludwigs-Universität, Freiburg, Germany. Peripheral blood mononuclear cells (PBMCs) were isolated from EDTA-anticoagulated blood by density gradient centrifugation. HLA-A*02:01 expression was confirmed by 4-digit HLA typing.

**TABLE 1:** Patient information^*a*^

Patient no.	Gender	Age (yr)	Viral load (IU/ml)	Therapy	NS3_1073_ epitope				NS3_1406_ epitope			
					Frequency of NS3_1073_-specific CD8^+^ T cells	Directly detectable T cells	% naive T cells	NS3_1073_ sequence	Frequency of NS3_1406_-specific CD8^+^ T cells	Directly detectable T cells	% naive T cells	NS3_1406_ sequence^*b*^
1	F	72	NA	Naive	6.08E−06	No	22.73	NA	1.26E−06	No	50.00	NA
2	M	52	<12	Nonresponder	1.74E−04	Yes	0.22	CINGVCWTV	9.29E−05	Yes	0.24	NA
3	M	52	865,280	Naive	1.31E−05	No	60.00	CINGVCWTV	7.42E−06	No	61.11	KLVALG**V**NAV
4	M	59	NA	Relapse	6.09E−06	No	25.00	CINGVCWTV	1.62E−05	No	12.12	KL**T**ALG**L**NAV
5	M	54	717,598	Naive	3.07E−05	No	11.76	CINGVCWTV	2.42E−05	No	73.47	KLVALGINAV
6	M	23	37,041	Naive	5.95E−06	No	30.30	CINGVCWTV	6.71E−06	No	71.88	KLVALG**V**NAV
7	M	34	1,406,062	Naive	2.39E−05	Yes	42.08	CINGVCWTV	2.36E−05	No	64.29	KL**T**A**M**GINAV
8	F	42	852,688	Nonresponder	3.99E−06	No	34.78	CINGVCWTV	5.03E−06	No	44.12	KLVALG**V**NAV
9	F	50	363,636	Naive	1.05E−04	Yes	23.93	CINGVCWTV	1.44E−04	Yes	9.41	KLVALG**V**NAV
10	F	46	521,509	Naive	1.51E−04	Yes	36.18	NA	7.45E−05	Yes	29.41	NA
11	M	43	382,481	Naive	4.10E−05	No	21.71	CINGVCWTV	6.19E−06	No	62.50	NA
12	M	44	<12	pegIFN, ribavirin, telaprevir	4.89E−04	Yes	2.87	CINGVCWTV	NA	NA	NA	NA
13	F	49	<12	pegIFN, ribavirin	6.25E−05	No	25.68	NA	1.52E−04	No	7.58	NA
14	M	50	738,876	Naive	1.99E−05	No	81.16	CINGVCWTV	NA	NA	NA	NA
	M	50	155	pegIFN, ribavirin, sofosbuvir	NA	NA	NA	NA	3.34E−06	No	70.59	KLVALGINAV
15	M	24	364,349	Naive	2.99E−05	No	69.53	CINGVCWTV	3.90E−05	Yes	14.89	KLVALGINAV
16	M	38	7,693,310	Naive	1.39E−05	No	27.03	NA	NA	NA	NA	NA
17	M	46	1,658,700	pegIFN, ribavirin, telaprevir	1.28E−05	No	25.93	NA	1.95E−05	No	9.09	NA

a M, male; F, female; NA, not available; pegIFN, polyethylene-glycosylated alpha interferon. Sequencing results are representative of those for the most common HCV variant. For patient 14, PBMCs from two different time points were used for the experiments.

b Amino acid variations in the HCV genotype 1a consensus sequence are shown in boldface.

### Tetramer enrichment.

Tetramer enrichment procedures were performed as described previously (2). Briefly, PBMCs were labeled with tetramers coupled to allophycocyanin (APC), and tetramer-bound cells were enriched using anti-APC magnetic beads and magnetic columns (both from Miltenyi Biotec, Bergisch Gladbach, Germany) according to the manufacturer's instructions. Preenriched, enriched, and depleted fractions were used for flow cytometric analysis. Between 10 and 1,366 tetramer-positive (tetramer^+^) CD8^+^ T cells were detectable after enrichment. The frequency of each epitope-specific T-cell population was determined using the calculation described by Alanio et al. (1).

### Antibodies and multiparametric flow cytometry.

The following monoclonal antibodies (MAbs) were used for phenotypic analysis: (i) anti-CD27-APC-eFluor780, anti-CD45RA-phycoerythrin (PE), and anti-CD127-eFluor450 (eBioscience, San Diego, CA); (ii) anti-CD3-Pacific Blue, anti-CD8-AmCyan, anti-CD8-V500, anti-CD11a-fluorescein isothiocyanate (FITC), anti-CD95-PE, anti-Ki67-FITC, and anti-CCR7-PE-Cy7 (BD Biosciences, Heidelberg, Germany); (iii) anti-CCR7-FITC (R&D Systems); and (iv) anti-PD-1-PE-Cy7 (BioLegend, San Diego, CA). 7-Aminoactinomycin D (7-AAD; Viaprobe; BD Biosciences) was used for the exclusion of dead cells. All samples were acquired using a FACSCanto II flow cytometer (BD Biosciences) and analyzed with FlowJo software (TreeStar Inc., Ashland, OR).

### Peptides and tetramers.

Peptides corresponding to HLA-A*02-restricted HCV-derived epitopes (NS3_1073_, CINGVCWTV; NS3_1406_, KLVALGINAV; variant, KLVALGVNAV), as well as a cytomegalovirus (CMV)-derived epitope (NLVPMVATV) and an influenza virus (Flu)-derived epitope (GILGFVFTL), were synthesized with free amino and carboxy termini by standard 9-fluorenylmethoxy carbonyl chemistry (Genaxxon Bioscience, Ulm, Germany). All peptides were dissolved in dimethyl sulfoxide and diluted in full medium (RPMI with 10% fetal bovine serum, 1% penicillin-streptomycin, and 1.5% 1 M HEPES; all from Life Technologies, Carlsbad, CA) according to previously reported protocols (9). Tetrameric peptide-MHC class I complexes were generated as described previously (10).

### Intracellular cytokine staining.

Intracellular cytokine staining procedures were performed as described previously (11). Briefly, cells were stimulated with peptide for 1 h in the presence of anti-CD107a-PE (BD Biosciences) and recombinant human interleukin-2 (rhIL-2; Hoffmann-La Roche, Basel, Switzerland), prior to addition of brefeldin A (GolgiPlug; BD Biosciences), and incubated for an additional 5 h before staining. Intracellular cytokine production was analyzed by using the following additional MAbs: (i) anti-CD8-APC-H7 and anti-tumor necrosis factor (anti-TNF)-PE-Cy7 (BD Biosciences) and (ii) anti-gamma interferon (anti-IFN-γ)-eFluor450 (eBioscience).

### MD-DCs and stimulation of CD8^+^ T cells.

Monocytes were purified, cultured, and matured to monocyte-derived dendritic cells (MD-DCs) as described previously (2). Briefly, CD14^+^ cells were selected using magnetic beads (Miltenyi Biotec) and cultured for 7 days in the presence of 2 ng/ml rhIL-4 and 10 ng/ml recombinant human granulocyte-macrophage colony-stimulating factor (both from Peprotech, Rocky Hill, NJ). MD-DCs were matured by addition of 10 ng/ml recombinant human TNF (eBioscience) on day 6. Matured MD-DCs were pulsed with a 10 μM concentration of the relevant peptide. In parallel, autologous CD8^+^ T cells were purified using magnetic beads (Miltenyi Biotec) and labeled with Pacific Blue succinimidyl ester (PBSE; Life Technologies) as described previously (2). Subsequently, MD-DCs and CD8^+^ T cells were cocultured in the presence of anti-CD28 MAb (0.5 μg/ml; BD Biosciences) at a ratio of 1:30 (MD-DCs/CD8^+^ T cells). rhIL-2 (20 U/ml) was added on days 4 and 8. On day 12, cells were used for phenotypic and functional analyses.

### DNA amplification and sequencing.

RNA was extracted from patient sera using a QIAamp viral RNA minikit (Qiagen, Hilden, Germany) and then subjected to reverse transcription (RT) and amplification with a One-Step RT-PCR kit (Qiagen), followed by nested PCR using a Titanium *Taq* PCR kit (Clontech, Mountain View, CA). The following primer combinations were used for DNA amplification and sequencing: (i) primers for RT and the first PCR, 5′-CRTCTGCTCCTGCTTGTGG (genomic location 2549; R = A/G) and 5′-ATCCGTGGARTGGCACTCR (genomic location 4294, R = A/G) for NS3_1073_ and 5′-GACAAAAACCARGYGGAGGG (genomic location 3516, R = A/G and Y = C/T) and 5′-GAGGACCTTCCCCAGYCC (genomic location 5735, Y = C/T) for NS3_1406_ and (ii) primers for nested PCR, 5′-ATGTGGCCTCTCCTCCTGC (genomic location 2740) and 5′-GCCACCTGGAAGCTCTGGG (genomic location 4004) for NS3_1073_ and 5′-ATAGCAGGGGYAGCCTGC (genomic location 3803, Y = C/T) and 5′-AGCACAGCCYGCGTCATAGC (genomic location 4905, Y = C/T) for NS3_1406_. Amplified DNA was purified using a QuickStep 2 PCR purification kit (EdgeBio, Gaithersburg, MD) and sequenced by GATC Biotech (Constance, Germany). The obtained bulk sequences were analyzed using the Sequencher (version 4.9) program (Gene Codes, Ann Arbor, MI).

### Statistics.

Statistical analysis was performed using GraphPad Prism (version 5) software (GraphPad Prism Software, Inc., La Jolla, CA). All tests were performed two-tailed and to a significance level of 95%. The statistical tests used are indicated in the figure legends (*, *P* < 0.05; **, *P* < 0.01; ***, *P* < 0.001; ****, *P* < 0.0001).

## RESULTS

### Enrichment of HCV-specific CD8^+^ T cells derived from chronically infected patients and healthy donors.

**FIG 1:**
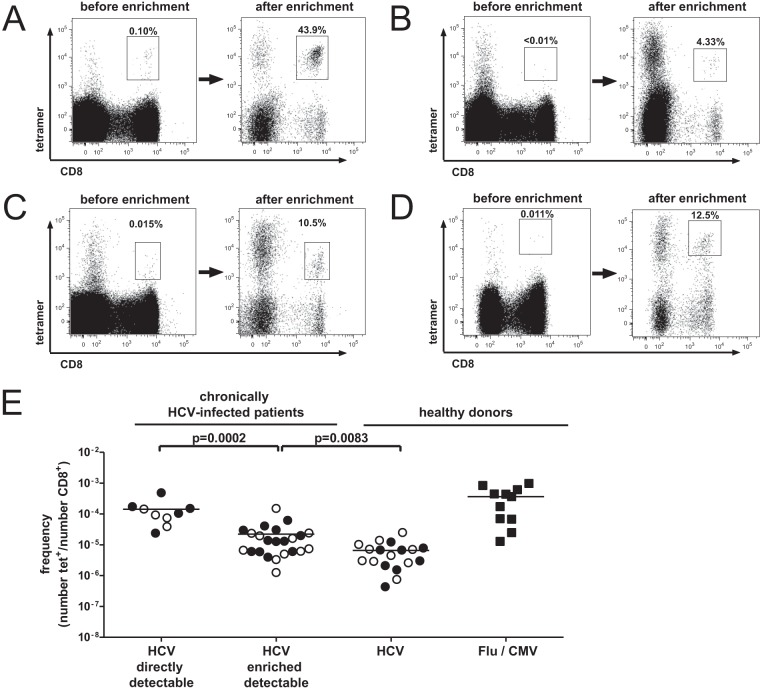
Tetramer enrichment and enumeration of HLA-A*02:01-restricted HCV-specific CD8^+^ T cells. (A to D) Representative flow cytometry plots showing tetramer stainings of PBMCs from patients with chronic HCV genotype 1a infection before and after magnetic bead enrichment. Numbers represent the frequency of tetramer^+^ (tet^+^) CD8^+^ T cells in total CD8^+^ T cells. (A) NS3_1406_-specific CD8^+^ T cells of patient 2 were detectable before tetramer enrichment. (B) NS3_1073_-specific CD8^+^ T cells of patient 1 were not detectable before tetramer enrichment. Patients 7 (C) and 15 (D) had *ex vivo* HCV-specific CD8^+^ T-cell responses near the limit of detection by conventional methods for NS3_1073_ (C) and NS3_1406_ (D), respectively. (E) Frequencies of CD8^+^ T cells specific for NS3_1073_ and NS3_1406_ after magnetic bead enrichment. The CD8^+^ T-cell responses of patients with chronic HCV infection were considered directly detectable if the *ex vivo* tetramer frequency was >0.01%. The frequencies of HCV-specific CD8^+^ T cells and Flu/CMV-specific CD8^+^ T cells in PBMCs from healthy donors are shown for comparison. The percentage of tetramer^+^ CD8^+^ T cells as a function of the total number of CD8^+^ T cells is indicated. Each symbol represents a CD8^+^ T-cell response to one epitope (filled circles, NS3_1073_; empty circles, NS3_1406_; filled squares, CMV/Flu). Horizontal bars represent mean values. *P* values were calculated using the Mann-Whitney U-test.

First, we analyzed the *ex vivo* frequencies of tetramer^+^ CD8^+^ T cells specific for two well-described HLA-A*02-restricted HCV-derived epitopes (NS3_1073_ and NS3_1406_). We analyzed 17 patients with chronic HCV genotype 1a infection (Table 1) and could detect HCV-specific CD8^+^ T cells in 9 of 32 cases (two epitopes were tested in 15 patients; one epitope was tested in 2 patients each). Next, we performed peptide-MHC class I tetramer enrichment for both epitopes using PBMCs obtained from the same patients. Representative plots are shown in Fig. 1A to D. Importantly, for all 32 CD8^+^ T-cell responses that were analyzed, virus-specific CD8^+^ T cells were detectable (Fig. 1E). For the purposes of this report, CD8^+^ T-cell populations that had an *ex vivo* frequency of >0.01% tetramer^+^ cells among CD8^+^ T cells were classified as “directly detectable,” whereas those that were detectable only after enrichment were termed “enriched detectable.” Thus, 9 CD8^+^ T-cell responses were directly detectable, whereas 23 were enriched detectable. Of note, several patients had a directly detectable response to one epitope but not to the other. As controls, we also enriched CD8^+^ T cells specific for HCV, Flu, and CMV from healthy donors. We observed clear differences in the frequency of virus-specific CD8^+^ T cells. The highest frequencies were observed for Flu- and CMV-specific CD8^+^ T cells in healthy donors. These were in a range similar to that described previously (1). Chronically HCV-infected patients with directly detectable HCV-specific CD8^+^ T-cell responses showed the second-highest frequencies of virus-specific CD8^+^ T cells. This is not surprising, given that these cells were all directly detectable *ex vivo* and showed average frequencies of approximately 10^−4^ tetramer^+^ CD8^+^ T cells after enrichment. This also correlates with the cutoff of the *ex vivo* tetramer analysis of 0.01%. Of note, two directly detectable HCV-specific CD8^+^ T-cell populations had lower frequencies, probably indicating that T-cell frequencies may be underestimated to some degree by the enrichment strategy (Fig. 1C and D). In contrast, enriched detectable HCV-specific CD8^+^ T cells showed a significantly lower frequency. Indeed, a frequency of 10^−4^ tetramer^+^ CD8^+^ T cells was observed in only a single case in this group. Healthy donors displayed the lowest frequencies of HCV-specific CD8^+^ T cells. The frequencies of NS3_1073_-specific and NS3_1406_-specific CD8^+^ T cells were similar in all groups (Fig. 1E). Of note, the high tetramer^+^ background in the CD8^−^ population (Fig. 1A to D) consisted mostly of CD19^+^ B cells, which have been described in the literature to bind to MHC class I tetramers (12). However, the CD8^+^ tetramer^+^ population was free of CD14^+^ monocytes, CD19^+^ B cells, CD16^+^ or CD56^+^ NK cells, and CD4^+^ T cells (data not shown). In conclusion, HCV-specific CD8^+^ T cells are present at very low frequencies in PBMCs during chronic HCV infection even in the absence of directly detectable *ex vivo* tetramer staining.

### Phenotypic characterization of enriched HCV-specific CD8^+^ T cells.

**FIG 2:**
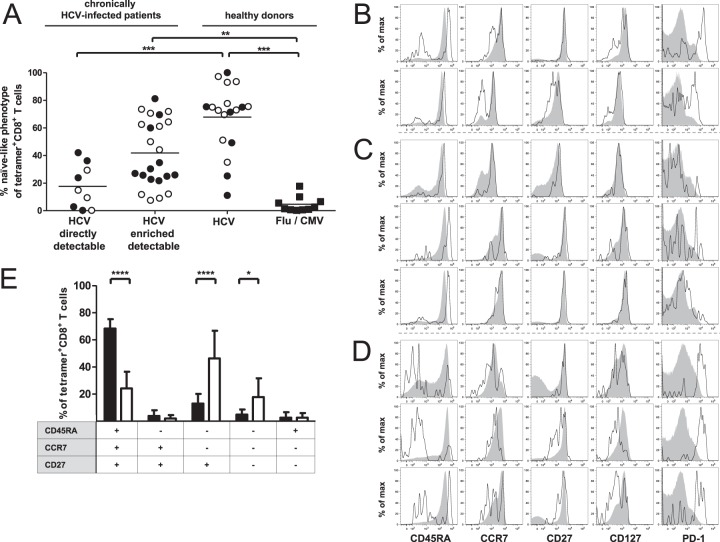
Phenotypic characterization of enriched HLA-A*02:01-restricted HCV-specific CD8^+^ T cells. (A) Frequency of tetramer^+^ CD8^+^ T cells with a naive-like phenotype (CD45RA^+^ CCR7^+^ CD27^+^) within the total enriched tetramer^+^ CD8^+^ T-cell population. Epitopes NS3_1073_ and NS3_1406_ from HCV in chronically HCV-infected patients were analyzed and compared to NS3_1073_ and NS3_1406_ as well as epitopes from CMV and Flu in healthy donors. Patients were grouped as described in the legend to Fig. 1E. Each symbol represents a CD8^+^ T-cell response to one epitope (filled circles, NS3_1073_; empty circles, NS3_1406_; filled squares, CMV/Flu). *P* values were calculated using one-way analysis of variance. Horizontal bars represent mean values. (B to D) Representative flow cytometry histograms showing the expression of CD45RA, CCR7, CD27, CD127, and PD-1 on enriched tetramer^+^ CD8^+^ T cells (black) and bulk CD8^+^ T cells (solid gray) in PBMCs from patients with chronic HCV infection. (B) Phenotypic characterization of directly detectable HCV-specific CD8^+^ T-cell responses with a high frequency of naive-like CD8^+^ T cells. NS3_1073_-specific CD8^+^ T-cell populations from patients 7 (top) and 10 (bottom) are displayed. (C) Enriched detectable HCV-specific CD8^+^ T-cell populations with a predominantly naive-like tetramer^+^ CD8^+^ T-cell phenotype are shown, from top to bottom, for NS3_1073_ in patient 14 and NS3_1406_ in patients 6 and 7, respectively. (D) Enriched detectable HCV-specific CD8^+^ T-cell populations with a predominant effector memory phenotype are shown, from top to bottom, for NS3_1073_ in patients 1, 5, and 6, respectively. (E) Bar diagram showing the coordinate expression of CD45RA, CCR7, and CD27 on tetramer^+^ CD8^+^ T cells from patients with enriched detectable HCV-specific CD8^+^ T-cell responses. The expression profile of the phenotypic markers is indicated below the diagram (13). Black bars, HCV-specific CD8^+^ T-cell populations with a predominantly (>60%) naive-like HCV-specific CD8^+^ T-cell population (*n* = 9); white bars, HCV-specific CD8^+^ T-cell populations with <60% naive-like CD8^+^ T cells (*n* = 15). Bars indicate mean values, and error bars indicate standard deviations. *P* values were calculated using two-way analysis of variance followed by Bonferroni's posttest. max, maximum.

**FIG 3:**
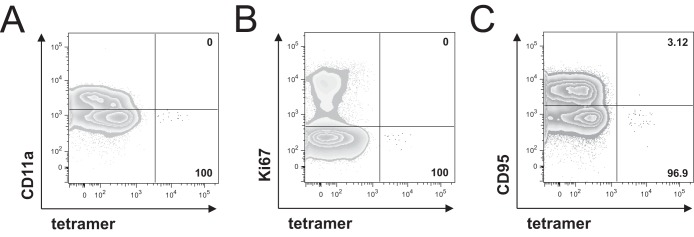
Phenotypic characterization of naive-like enriched HLA-A*02:01-restricted HCV-specific CD8^+^ T cells. Representative flow cytometry plots showing the expression of CD11a (A), Ki67 (B), and CD95 (C) on naive-like (CD45RA^+^ CCR7^+^ CD27^+^ CD127^+^) CD8^+^ T cells in PBMCs from patients 14 (NS3_1406_) (A), 3 (NS3_1073_) (B), and 8 (NS3_1406_) (C) with chronic HCV infection. Naive-like tetramer^+^ CD8^+^ T cells (black) are shown overlaid on bulk CD8^+^ T cells (gray). Numbers represent the frequency of expression on naive-like tetramer^+^ CD8^+^ T cells.

Next, we performed a detailed phenotypic analysis of the enriched virus-specific CD8^+^ T cells by using multiparametric flow cytometry to determine the coordinate expression of various surface markers. Naive CD8^+^ T cells are characterized by the coexpression of CD45RA, CCR7, and CD27 (13). As shown in Fig. 2A, Flu and CMV-specific CD8^+^ T cells rarely displayed a naive-like phenotype, indicating that these cells had been primed *in vivo* and represent memory populations. As expected and described previously, the majority of HCV-specific CD8^+^ T cells obtained from healthy donors displayed a naive-like phenotype (2). However, nonnaive HCV-specific CD8^+^ T cells were observed in some seronegative individuals; this finding is consistent with that of previous studies and likely related to specific T-cell receptor cross recognition of alternative priming antigens (14–17). Furthermore, most directly detectable HCV-specific CD8^+^ T-cell responses showed a predominantly antigen-experienced phenotype. Only four directly detectable HCV-specific CD8^+^ T-cell responses showed relatively high frequencies of cells with naive-like phenotypes (23.93% to 42.08%; Fig. 2A and B). This may be linked, for example, to incomplete priming of the original naive CD8^+^ T-cell population. Enriched detectable HCV-specific CD8^+^ T cells, however, showed a more heterogeneous differentiation profile (Fig. 2A), ranging from a predominantly naive-like CD45RA^+^ CCR7^+^ CD27^+^ phenotype with high levels of CD127 expression (Fig. 2C and E) to an effector memory CD45RA^−^ CCR7^−^ CD27^+^ phenotype with high expression levels of the exhaustion marker PD-1 (Fig. 2D and E). Low CD11a expression and a lack of the proliferation marker Ki67 further indicate a naive phenotype of these cells (Fig. 3A and B). In addition, we analyzed CD95 expression, which has been suggested to distinguish truly naive CD95^−^ T cells and CD95^+^ stem cell memory T (T_SCM_) cells (18, 19). The absence of CD95 expression points toward a naive status of HCV-specific CD8^+^ T cells with a CD45RA^+^ CCR7^+^ CD27^+^ phenotype (Fig. 3C). In sum, small numbers of HCV-specific CD8^+^ T cells with an effector memory or naive-like phenotype are present at levels below the limit of detection by conventional tetramer staining in patients with chronic HCV infection. Notably, both effector memory and naive-like CD8^+^ T cells specific for the NS3_1073_ and NS3_1406_ epitopes could be found in individual patients (Table 1). This observation suggests that neither low viral titers nor specific host genetic features in isolation can explain the presence of naive-like HCV-specific CD8^+^ T cells during chronic HCV infection.

### Functional capacities of naive-like CD8^+^ T-cell populations derived from patients with chronic HCV infection.

**FIG 4:**
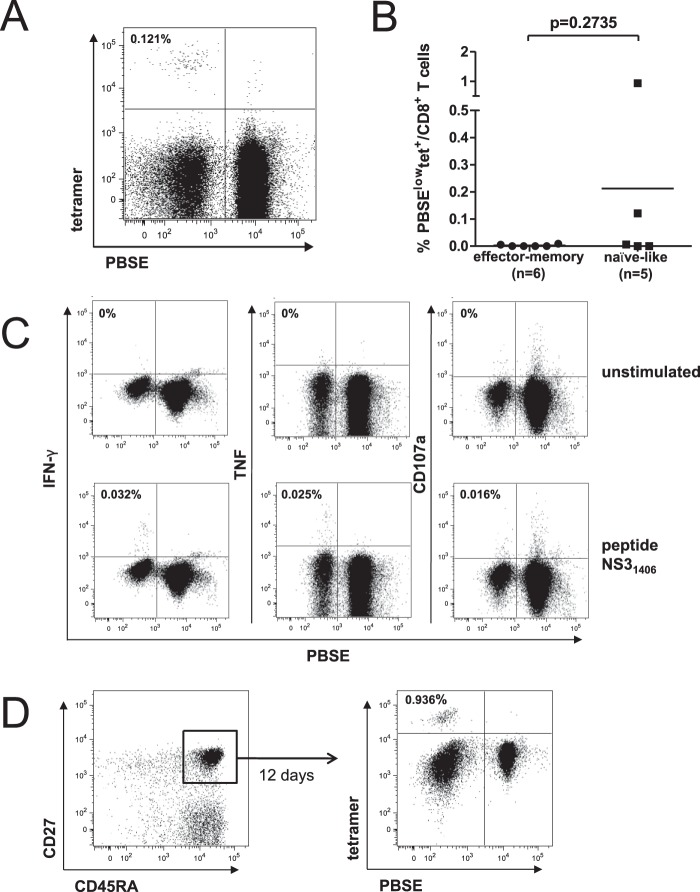
Proliferation of patient-derived enriched detectable HCV-specific CD8^+^ T cells after stimulation with autologous peptide-pulsed MD-DCs. (A) Representative flow cytometry plot showing the proliferation of NS3_1406_-specific CD8^+^ T cells with a predominantly naive-like phenotype from patient 7 with chronic HCV infection. CD8^+^ T cells were labeled with PBSE and then stimulated for 12 days with NS3_1406_ peptide-pulsed autologous MD-DCs. Proliferation was measured by PBSE dilution. The frequency of PBSE^low^ tetramer^+^ CD8^+^ T cells in total CD8^+^ T cells is indicated. (B) Frequency of tetramer^+^ CD8^+^ T cells after 12 days of stimulation with NS3_1073_ or NS3_1406_ peptide-pulsed autologous MD-DCs. Only patients with enriched detectable epitope-specific CD8^+^ T-cell populations were analyzed. HCV-specific CD8^+^ T-cell responses with >60% naive-like CD8^+^ T cells were grouped as naive-like. Each dot represents a CD8^+^ T-cell response to one epitope. *P* values were calculated using the Mann-Whitney U test. Horizontal bars represent mean values. (C) Representative flow cytometry plots showing the production of IFN-γ and TNF as well as CD107a mobilization of the *in vitro*-expanded HCV-specific CD8^+^ T cells from patient 7. Results for unstimulated (top) and NS3_1406_ peptide-stimulated (bottom) samples are shown. Numbers indicate the percentage of marker-positive PBSE^low^ CD8^+^ T cells after subtraction of the background. (D) CD8^+^ T cells were enriched from patient-derived PBMCs by using magnetic beads and further sorted by flow cytometry on the basis of CD45RA^+^ and CD27^+^ coexpression (left). The purified CD8^+^ CD45RA^+^ CD27^+^ cells were labeled with PBSE and stimulated with NS3_1406_ peptide-pulsed autologous MD-DCs for 12 days (right). Patient 6 was selected for the experiment whose results are shown on the basis of the presence of NS3_1406_-specific CD8^+^ T cells with a predominantly naive-like phenotype during chronic HCV infection.

Subsequently, we analyzed the proliferative capacity of enriched detectable HCV-specific CD8^+^ T-cell populations. Naive CD8^+^ T cells classically retain the ability to proliferate vigorously in response to appropriate priming. To address whether naive-like HCV-specific CD8^+^ T cells can be primed and proliferate in the chronic phase of infection, we stimulated patient-derived CD8^+^ T cells with peptide-pulsed autologous monocyte-derived dendritic cells (MD-DCs) for 12 days and performed a combined phenotypic and functional analysis. As shown for one representative patient (Fig. 4A), two of five HCV-specific CD8^+^ T-cell populations with a predominantly (>60%) naive-like phenotype were able to expand upon specific stimulation (Fig. 4B). Functional analyses revealed that the expanded CD8^+^ T-cell populations were able to produce both IFN-γ and TNF as well as to weakly mobilize CD107a (Fig. 4C). To confirm that naive-like epitope-specific CD8^+^ T cells contributed directly to the expanded response *in vitro*, we sorted naive-like CD8^+^ T cells (CD45RA^+^ CD27^+^) from patient-derived PBMCs prior to stimulation with peptide-pulsed autologous MD-DCs. As shown in Fig. 4D, these sorted cells were able to proliferate, suggesting that in some patients with chronic HCV infection, naive-like HCV-specific CD8^+^ T cells can expand *in vitro*. In contrast, HCV-specific CD8^+^ T-cell populations with an effector memory phenotype were unable to proliferate upon specific stimulation (Fig. 4B). This lack of proliferation in combination with the observed high expression of PD-1 (Fig. 2D) may indicate that these cells are in a state of exhaustion (20, 21). Even though we detected small amounts of naive-like HCV-specific CD8^+^ T cells in predominantly effector memory phenotype populations (Fig. 2A), these were unable to proliferate *in vitro*.

### Viral sequence analysis of corresponding epitopes.

**FIG 5:**
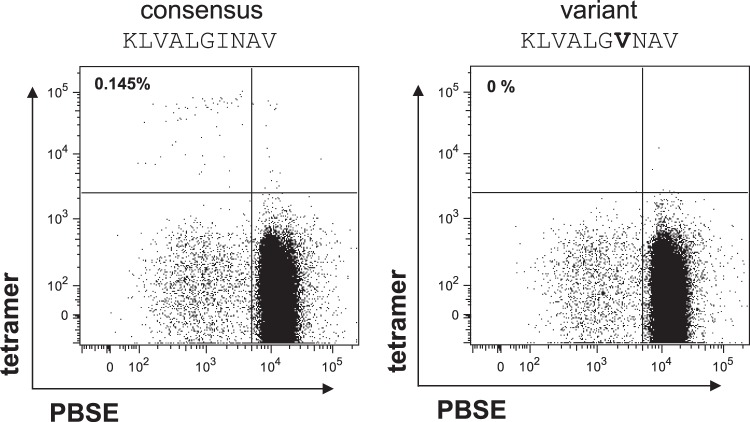
Proliferation of patient-derived enriched detectable HCV-specific CD8^+^ T cells occurs only in response to the consensus epitope. MD-DCs from patient 6 were pulsed either with the NS3_1406_ consensus sequence or with the autologous variant and used for *in vitro* priming. The frequency of PBSE^low^ tetramer^+^ CD8^+^ T cells in total CD8^+^ T cells is indicated. The altered amino acid is shown in boldface.

The antigen recognition and, thus, the phenotype of HCV-specific CD8^+^ T cells can be affected by the presence of sequence variations in viral epitopes, as previously described (4). Thus, we analyzed bulk viral sequences corresponding to the epitopes NS3_1073_ and NS3_1406_ from patients with enriched detectable HCV-specific CD8^+^ T-cell responses (Table 1). In our cohort, all patients showed the genotype 1a consensus sequence for the NS3_1073_ epitope, whereas most patients showed viral sequence variations for the NS3_1406_ epitope. This was independent of the naive-like or effector memory phenotype of HCV-specific CD8^+^ T cells. Since we were able to expand only two out of five HCV-specific CD8^+^ T-cell populations with a predominantly naive-like phenotype, we next investigated whether there is a correlation between sequence variations in the corresponding viral epitope and the proliferative capacity of naive-like HCV-specific CD8^+^ T cells, as observed in Fig. 4B. In HCV-specific CD8^+^ T-cell epitopes with the genotype 1a consensus sequence, we did not observe HCV-specific CD8^+^ T-cell proliferation. In contrast, in the two patients where we found *in vitro* expansion of naive-like HCV-specific CD8^+^ T cells, viral sequence variations in the corresponding CD8^+^ T-cell epitopes were detectable (Table 2). These results suggest that the sequence variations may not have developed during chronic infection (viral escape) but may have already been present in the infecting virus. To confirm this point, we analyzed the effect of the viral sequence variations on HCV-specific CD8^+^ T-cell priming. Patient 6 showed a high frequency of naive-like NS3_1406_-specific CD8^+^ T cells and sequence variation in the NS3_1406_ epitope. We pulsed MD-DCs from this patient with the consensus as well as the autologous variant sequence of NS3_1406_ and used these sequences to stimulate the naive-like HCV-specific CD8^+^ T cells. Importantly, proliferation of naive-like NS3_1406_-specific CD8^+^ T cells could only be observed using the consensus sequence and not the autologous variant sequence, indicating a lack of recognition of the variant sequence (Fig. 5). This finding suggests that functional, naive-like HCV-specific CD8^+^ T cells directed against the consensus epitope may persist only in patients infected with viral variants.

**TABLE 2:** Viral sequence variations in relation to proliferative capability of naive-like HCV-specific CD8^+^ T cells^*a*^

Outcome of priming	NS3_1073_ sequence	NS3_1406_ sequence
	CINGVCWTV	KLVALGINAV
Proliferation of naive-like T cells		------V--- (pt. 6)
		--T-M----- (pt. 7)
Lack of proliferation of naive-like T cells	--------- (pt. 14)	---------- (pt. 14)
		---------- (pt. 5)

a Autologous bulk viral sequences that correspond to the five naive-like HCV-specific CD8^+^ T-cell responses from Fig. 4B are shown. For each epitope, the sequence in each line represents the sequence obtained from one patient; hyphens represent sequence homology to the genotype 1a consensus sequence. Numbers in parentheses correspond to patient (pt.) numbers from Table 1.

## DISCUSSION

In this study, we set out to detect and characterize HCV-specific CD8^+^ T cells derived from chronically HCV genotype 1a-infected patients without HCV-specific CD8^+^ T-cell responses detectable by conventional tetramer staining. Therefore, we used an MHC class I tetramer enrichment protocol that allows the detection of epitope-specific CD8^+^ T-cell populations present at very low numbers (1). We decided to analyze two well-described HLA-A*02:01-restricted CD8^+^ T-cell epitopes, NS3_1073_ and NS3_1406_, which are frequently recognized (8, 22). Our results show that by using the tetramer enrichment strategy, HCV-specific CD8^+^ T-cell populations are detectable in all patients with chronic HCV infection, even in those without detectable responses *ex vivo*. Importantly, in chronically infected patients with enriched detectable HCV-specific CD8^+^ T-cell responses, we observed heterogeneous T-cell phenotypes. Nine of 23 enriched detectable HCV-specific CD8^+^ T-cell responses displayed a naive-like phenotype (Fig. 2A). The low frequencies of naive-like HCV-specific CD8^+^ T cells in HCV-infected patients are in line with the low numbers of these cells in healthy donors (2). The other 14 HCV-specific CD8^+^ T-cell responses displayed an effector memory phenotype with a high expression of the inhibitory receptor PD-1, a marker of CD8^+^ T-cell exhaustion (4). It has been described that exhausted HCV-specific CD8^+^ T cells can be deleted from the T-cell pool (21). This in turn may also explain the low numbers of epitope-specific CD8^+^ T cells in many chronically infected patients. Thus, both the naive-like and the exhausted effector memory phenotype may explain the absence of *ex vivo*-detectable HCV-specific CD8^+^ T-cell counts in these chronically HCV-infected patients.

To the best of our knowledge, the presence of naive-like virus-specific CD8^+^ T cells despite ongoing viral replication has not been described previously in humans. The presence of naive-like HCV-specific CD8^+^ T-cell populations after enrichment may be explained by an impaired priming of these CD8^+^ T cells during acute infection, a process that we sought to reproduce using an *in vitro* priming assay. Of note, we observed a heterogeneous effect of *in vitro* priming in HCV-specific CD8^+^ T-cell populations with a predominantly (>60%) naive-like phenotype. Indeed, proliferation of HCV-specific CD8^+^ T cells was observed in only two of five naive-like HCV-specific CD8^+^ T-cell populations. Thus, the possibility of a functional impairment of naive-like HCV-specific CD8^+^ T cells cannot be excluded in all cases. However, viral sequence variations in the corresponding epitopes may also alter antigen recognition by CD8^+^ T cells (23). Indeed, analysis of the viral sequences corresponding to the CD8^+^ T-cell epitopes NS3_1073_ and NS3_1406_ indicated a correlation between viral sequences and the proliferative capacity of HCV-specific CD8^+^ T-cell populations with a naive-like phenotype. Due to the limited number of patients with which we could perform these analyses, this correlation should be confirmed with a larger patient cohort. Naive-like HCV-specific CD8^+^ T-cell populations targeting consensus viral sequences could not proliferate *in vitro*. This may indeed suggest a functional impairment of naive-like HCV-specific CD8^+^ T cells targeting consensus sequences in some patients, preventing their priming during acute infection. Importantly, this is not a general CD8^+^ T-cell defect but rather an epitope-specific phenomenon, since patient 5 showed an effector memory phenotype for NS3_1073_ but a naive-like CD8^+^ T-cell population for NS3_1406_ that could not expand *in vitro*, arguing against a major role of host-specific genetic features or low viral loads. One explanation for this functional impairment may be a reduced avidity of HCV-specific CD8^+^ T cells for their cognate antigen mediated by a loss of high-avidity clones from the CD8^+^ T-cell repertoire. This could lead to reduced antigen recognition (24). A low functional avidity may also explain why *in vitro* priming did not result in proliferation of the remaining naive-like HCV-specific CD8^+^ T cells in patients with effector memory phenotype responses. However, further research will be required to address this important question, since we could not analyze the functional avidity of enriched detectable HCV-specific CD8^+^ T cells because of insufficient cell numbers.

In contrast, in two patients where naive-like HCV-specific CD8^+^ T cells were able to proliferate *in vitro*, variant sequences were obtained for the corresponding CD8^+^ T-cell epitopes (Table 2). Importantly, this is, in our opinion, not consistent with viral escape mutations, since we did not observe effector memory CD8^+^ T-cell responses toward these epitopes, arguing against immune-mediated selection pressure. Thus, viral sequence variations in these epitopes were likely present in the original infecting virus. Of note, naive-like NS3_1406_-specific CD8^+^ T cells from one of these patients proliferated only in response to the genotype 1a consensus sequence and not in response to the autologous variant sequence (Fig. 5). Thus, functionally competent naive-like HCV-specific CD8^+^ T cells likely persisted in these patients due to the absence of matching antigen. In conclusion, these results may suggest that some HCV-specific CD8^+^ T-cell populations have never been primed during early infection. This lack of CD8^+^ T-cell priming may be mediated either extrinsically by a variant infecting virus or intrinsically by a functional impairment of CD8^+^ T cells. To our knowledge, this is the first description that, in addition to T-cell exhaustion and viral escape mutations, a lack of CD8^+^ T-cell priming may limit the efficacy of HCV-specific CD8^+^ T cells.
